# Swainson’s Thrushes do not show strong wind selectivity prior to crossing the Gulf of Mexico

**DOI:** 10.1038/s41598-017-14668-3

**Published:** 2017-10-27

**Authors:** Rachel T. Bolus, Robert H. Diehl, Frank R. Moore, Jill L. Deppe, Michael P. Ward, Jaclyn Smolinsky, Theodore J. Zenzal

**Affiliations:** 1 U. S. Geological Survey, Northern Rocky Mountain Science Center, Bozeman, MT 59715 USA; 2 University of Illinois at Urbana-Champaign, Department of Natural Resources and Environmental Sciences, Urbana, IL 61801 USA; 3 Southern Utah University, Department of Biology, Cedar City, UT 84720 USA; 4 The University of Southern Mississippi, Department of Biological Sciences, Hattiesburg, MS 39406 USA; 5 Eastern Illinois University, Department of Biology, Charleston, IL 61920 USA; 6 University of Delaware, Department of Entomology and Wildlife Ecology, Newark, DE 19716 USA

## Abstract

During long-distance fall migrations, nocturnally migrating Swainson’s Thrushes often stop on the northern Gulf of Mexico coast before flying across the Gulf. To minimize energetic costs, trans-Gulf migrants should stop over when they encounter crosswinds or headwinds, and depart with supportive tailwinds. However, time constrained migrants should be less selective, balancing costs of headwinds with benefits of continuing their migrations. To test the hypotheses that birds select supportive winds and that selectivity is mediated by seasonal time constraints, we examined whether local winds affected Swainson’s Thrushes’ arrival and departure at Ft. Morgan, Alabama, USA at annual, seasonal, and nightly time scales. Additionally, migrants could benefit from forecasting future wind conditions, crossing on nights when winds are consistently supportive across the Gulf, thereby avoiding the potentially lethal consequences of depleting their energetic reserves over water. To test whether birds forecast, we developed a movement model, calculated to what extent departure winds were predictive of future Gulf winds, and tested whether birds responded to predictability. Swainson’s Thrushes were only slightly selective and did not appear to forecast. By following the simple rule of avoiding only the strongest headwinds at departure, Swainson’s Thrushes could survive the 1500 km flight between Alabama and Veracruz, Mexico.

## Introduction

Long-distance avian migrants have evolved behaviours to cope with the vagaries of winds^1,2^. Tailwinds can support the energetic work of transport^3^, yet energy depleting headwinds or disorienting crosswinds may appear and persist in any given year, part of the season, or flight^4,5^. Confronted with variable winds, migrating birds often time and orient their flights to optimize available wind support^6–10^.

Migratory birds should select winds depending on the consequences of selectivity, which are mediated by whether individuals are time constrained and the availability of stopover sites (i.e., resting and refueling sites). During the fall, time constraints may include deteriorating conditions or resources on the breeding grounds and along the migratory route^5^ or the need to arrive quickly to the wintering grounds to secure high-quality territories^11–13^. If time constrained, migrants should be more selective of supportive winds that can increase overall migration speed, provided supportive winds occur frequently. However, if supportive winds are rare or typically weak and/or migrants have to wait too long, they may be decreasingly selective and fly in headwinds as continuing to migrate even small distances each night will cause less delay than continuing to stop over^14,15^. At the smaller nightly timescale, choosing to fly with headwinds during a short flight when stopover sites are readily available should minimally affect fitness, as birds can land and wait for better conditions at any time^16^. In contrast, wind selectivity should be important for birds committed to long-distance flights with few or no opportunities for foraging or landing (e.g., crossing the Gulf of Mexico); in this case the consequences of choosing non-supportive winds can be severe, including extreme physiological stress or death^17,18^.

Even if migrants benefit from selecting supportive winds, local weather cues may not be predictive of future wind conditions. However, despite this disconnect there is some evidence that birds can forecast future wind conditions, especially in systems where wind conditions are similar over broad spatial extents and timescales^19–22^. For example, Reed Warblers (*Acrocephalus scirpaceus*) departing Falsterbo, Sweden used barometric pressure rather than local winds as the cue for migratory departure, suggesting that their decisions were affected by cues that predicted broad synoptic weather patterns rather than local conditions^23^. If possible, forecasting should be an adaptive strategy for long flights over large areas of unsuitable habitat (e.g., deserts, large water bodies), as future weather may affect survival more than local weather.

**Figure 1:**
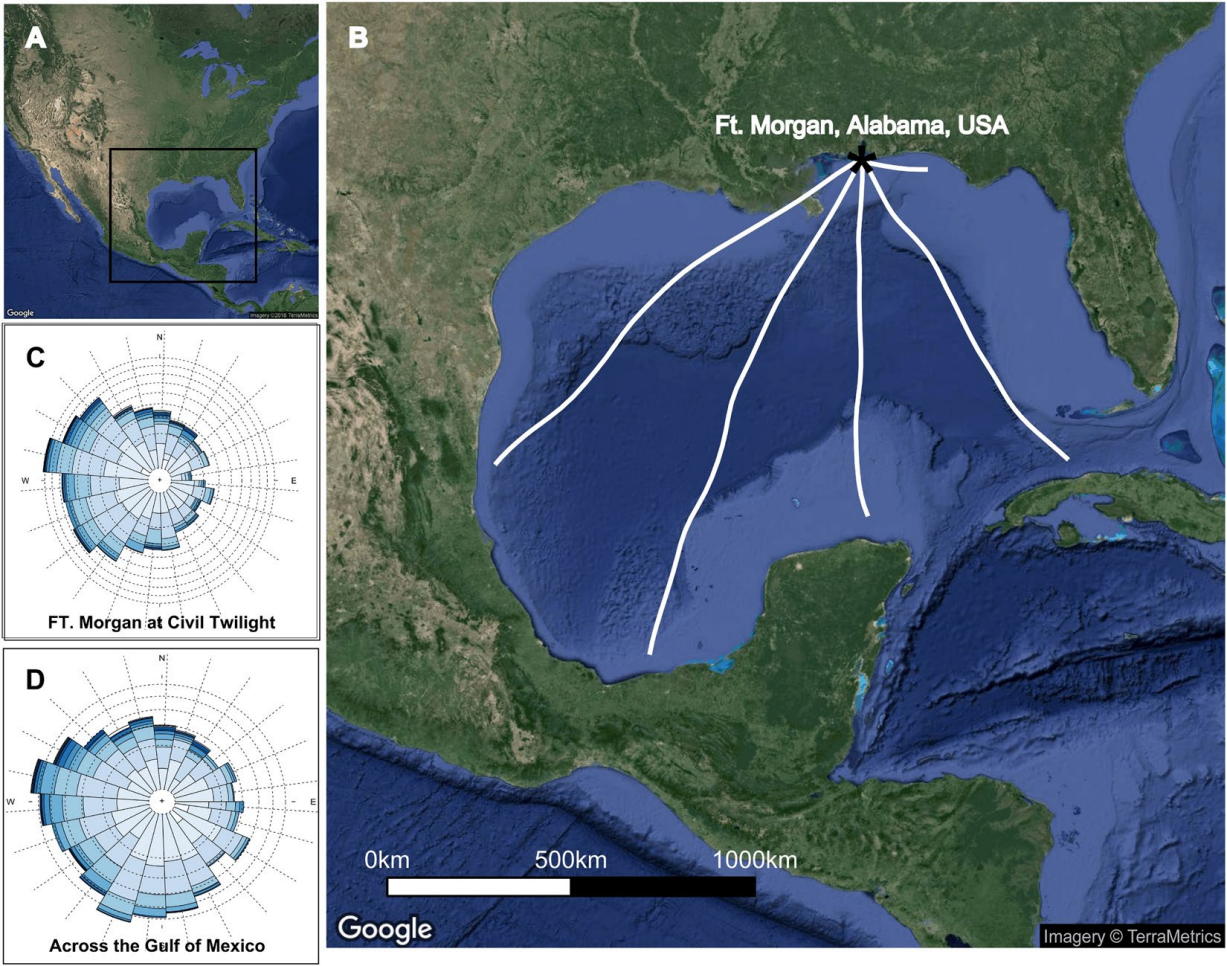
Study System: The Gulf of Mexico. (**A**) The Gulf of Mexico is in North America; two-thirds of North American avian migrants encounter it during fall migration. (**B**) In Ft. Morgan, we captured migrant landbirds at our long-term banding station (1992–2012) and recorded radio-tagged Swainson’s Thrush departure behaviours using an automated telemetry array (2008–2012). To estimate whether birds could forecast future wind conditions across the Gulf, we simulated tracks each night between 1 Sep and 31 Oct with five potential departure directions: 120°, 150°, 180°, 210°, and 240°. The predicted tracks varied depending on nightly wind conditions, but estimated the wind conditions across the extent of the Gulf that migrants would likely experience. (**C**) Wind rose of wind directions at Ft. Morgan at civil twilight, the time of departure for most nocturnal migrants (darker blue indicates higher speeds, with an interval of 2 m·s^−1^). The mean wind direction was 231°. (**D**) Wind rose of mean wind directions across all five tracks across the Gulf. The mean wind direction was 273°. Map data from TerraMetrics and access through Google.

We describe wind variation and evaluated migrant selectivity for wind conditions at different temporal scales for migratory Swainson’s Thrushes (*Catharus ustulatus*) at the population and individual levels. First, we used long-term capture data to describe wind selectivity during fall migration of thrush populations at annual, seasonal and nightly time scales based on thrushes’ decisions to arrive (i.e., interrupt flight by landing to rest and/or refuel) at a stopover site on the northern Gulf of Mexico in Ft. Morgan, Alabama, USA (Fig. 1). We predicted that migratory Swainson’s Thrushes would be selective of the winds they chose to cross the Gulf, and therefore would be more likely to stop on the coast when faced with non-supportive headwinds or crosswinds. Second, we used automated radio telemetry data to examine nightly wind selectivity using departure behaviour of individual Swainson’s Thrushes that initiated flights across the Gulf. Based on our hypothesis that migratory thrushes should demonstrate wind selectivity, we expected that Swainson’s Thrushes should depart with supportive tailwinds. Third, we hypothesized that Swainson’s Thrushes are able to forecast future supportive winds, and were more likely to depart on nights when local wind conditions were predictive of future wind conditions over the Gulf. Finally, we also used Pennycuick’s^24^ flight energetic model to estimate Swainson’s Thrushes’ potential flight ranges to enhance our understanding of the challenges associated with over-water flights across the Gulf of Mexico and the significance of wind selectivity in this system.

## Results

### Stopover decisions

Overall, mean and standard deviation nightly tailwinds during fall migration at Ft. Morgan were −0.60 ± 4.4 m·s^−1^ for all nights (1992–2012; n = 1,281) and −0.14 ± 3.62 m·s^−1^ for the subset of nights that the banding station was open the following day (n = 1,074, all reported values are means +/− standard deviation unless stated otherwise). Mean nightly Ft. Morgan winds were supportive 50% of these nights. Also, winds typically worsened over the course of a night. The mean slope of tailwinds over a night was −0.13 ± 0.26 m·s^−1^ per hour.

**Figure 2:**
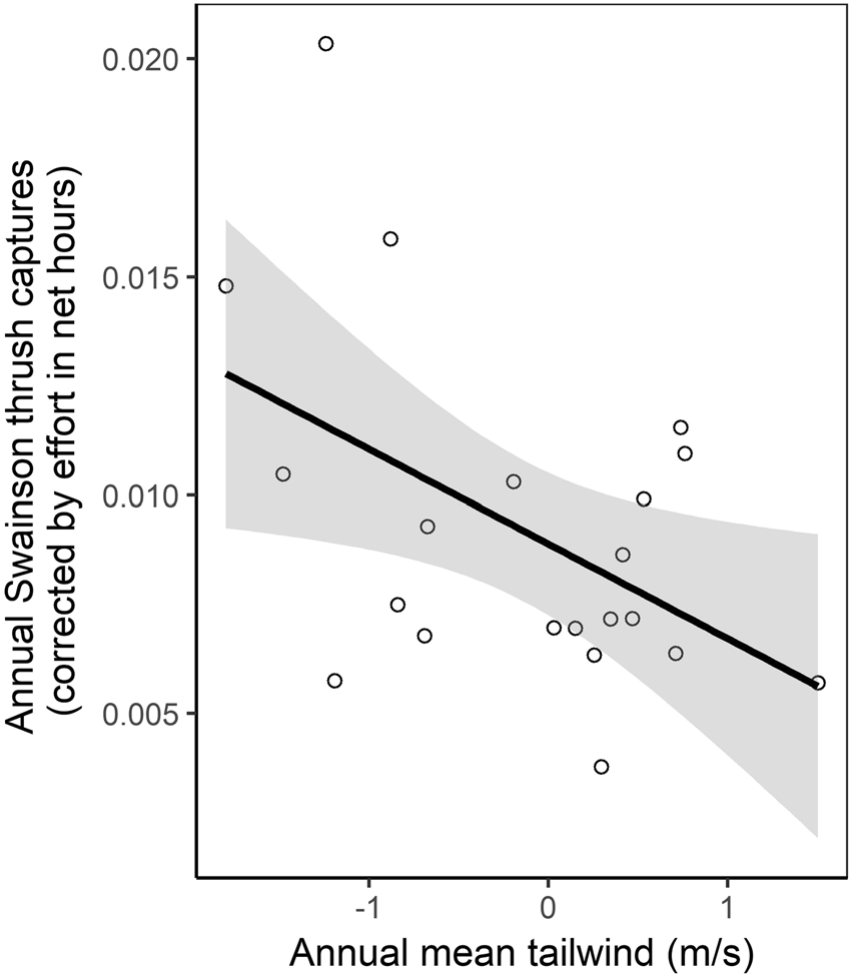
There was a significant negative relationship between annual mean nightly wind profits and capture rate of Swainson’s Thrushes on Ft. Morgan Peninsula. The shaded area delineates a 95% CI.

Between 1992 and 2012, 1,352 Swainson’s Thrushes were captured at Ft. Morgan with an annual mean of 64 ± 26 birds (0.009 ± 0.004 birds·net hour^−1^). Birds arrived between 4 September and 30 October, with a median capture date of 3 October and an interquartile range of 12 days (Supplementary Fig. S1). As mist-netting activities ended on 31 October, we may have missed some late migrants, but the tapering off of the phenological distribution at this time suggested that our sampling period largely matched the Swainson’s Thrush migration period in this region. There was a significant negative relationship between the number of Swainson’s Thrushes captured annually (corrected by sampling effort) and the mean annual tailwind (Fig. 2; F_1;19_ = 5.8, adjusted R^2^ = 0.19, p = 0.026), and mean annual tailwind was positively correlated with the annual proportion of nights with supportive winds (r = 0.92, df = 19, p < 0.0001).

**Figure 3:**
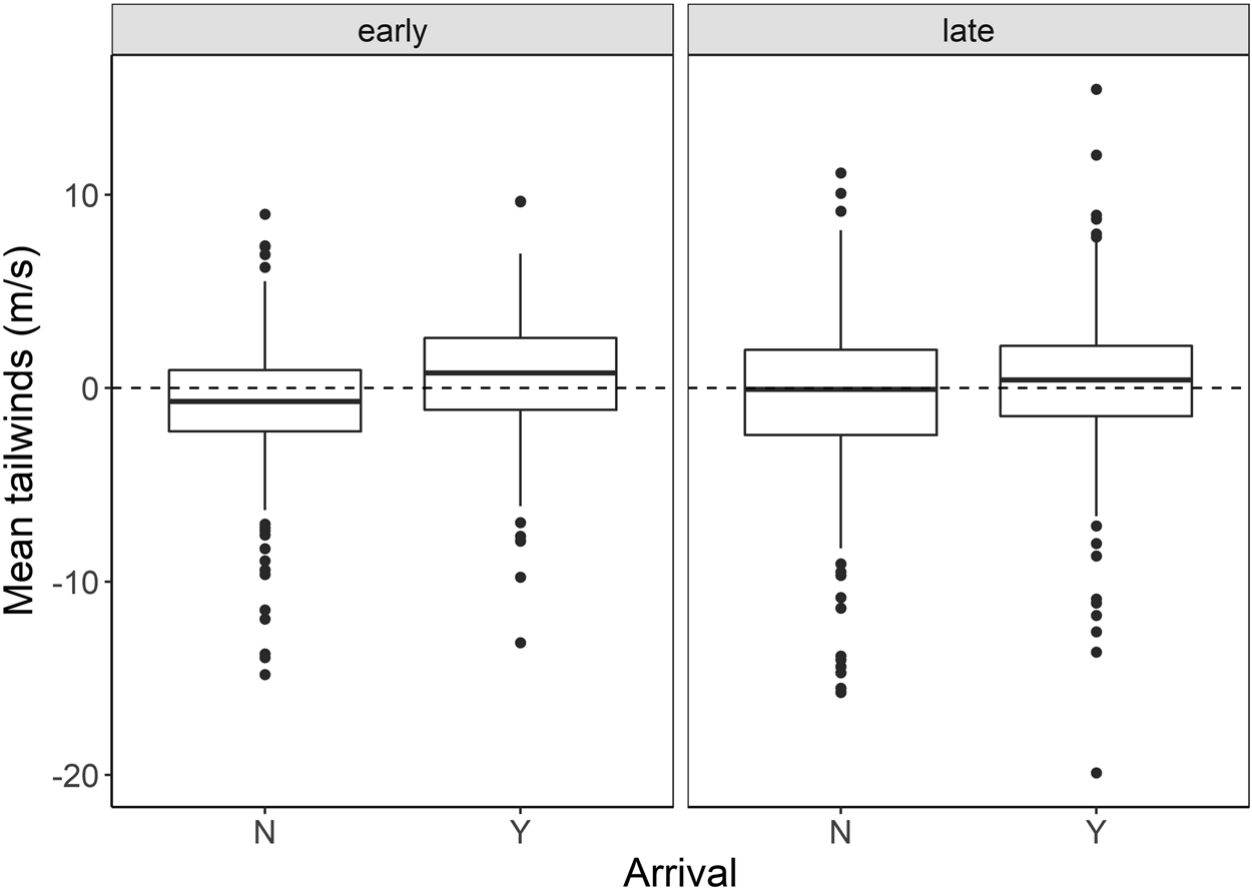
There was a significant interaction between tailwind and period (early or late in the season) on the probability of arrival at Ft. Morgan for stopover. Specifically, birds were more selective earlier in the season, arriving for stopover on nights with a mean tailwind of 0.64 ± 3.05 m·s^−1^ compared to −0.82 ± 3.03 m·s^−1^ on nights when they did not arrive, an effect size of 1.46 m·s^−1^. In comparison, in the late season, birds arrived for stopover on nights with a mean tailwind of 0.25 ± 3.93 m·s^−1^ compared to −0.45 ± 4.28 m·s^−1^ on nights when they did not arrive, an effect size of 0.7 m·s^−1^.

The model testing the effects of period, tailwind, and their interactions on probability of stopover was significantly different than a null model (χ^2^ = 38.9, df = 3, p < 0.0001, Table 1); variables had low multicollinearity (VIF_period_ = 1.00, VIF_tailwind_ = 3.02, VIF_period_ × _tailwind_ = 3.02). The probability of arriving for stopover was more likely on nights that had better tailwinds. There was also a significant interaction between tailwinds and period: birds were more selective of tailwinds in the early half of the migration season (Fig. 3).

**Table 1:** Logistic regression covariates of analysis on the probability of arrival for stopover at Ft. Morgan over 1074 nights, in relation to time of season (period), mean tailwind, and the interaction between period and tailwind.

	Estimate ± S. E.	p
Period (Late)	0.168 ± 0.086	0.178
Mean tailwind	0.170 ± 0.032	< 0.001
Period × Mean tailwind	−0.128 ± 0.039	0.001
Intercept	−0.220 ± 0.086	0.011

**Figure 4:**
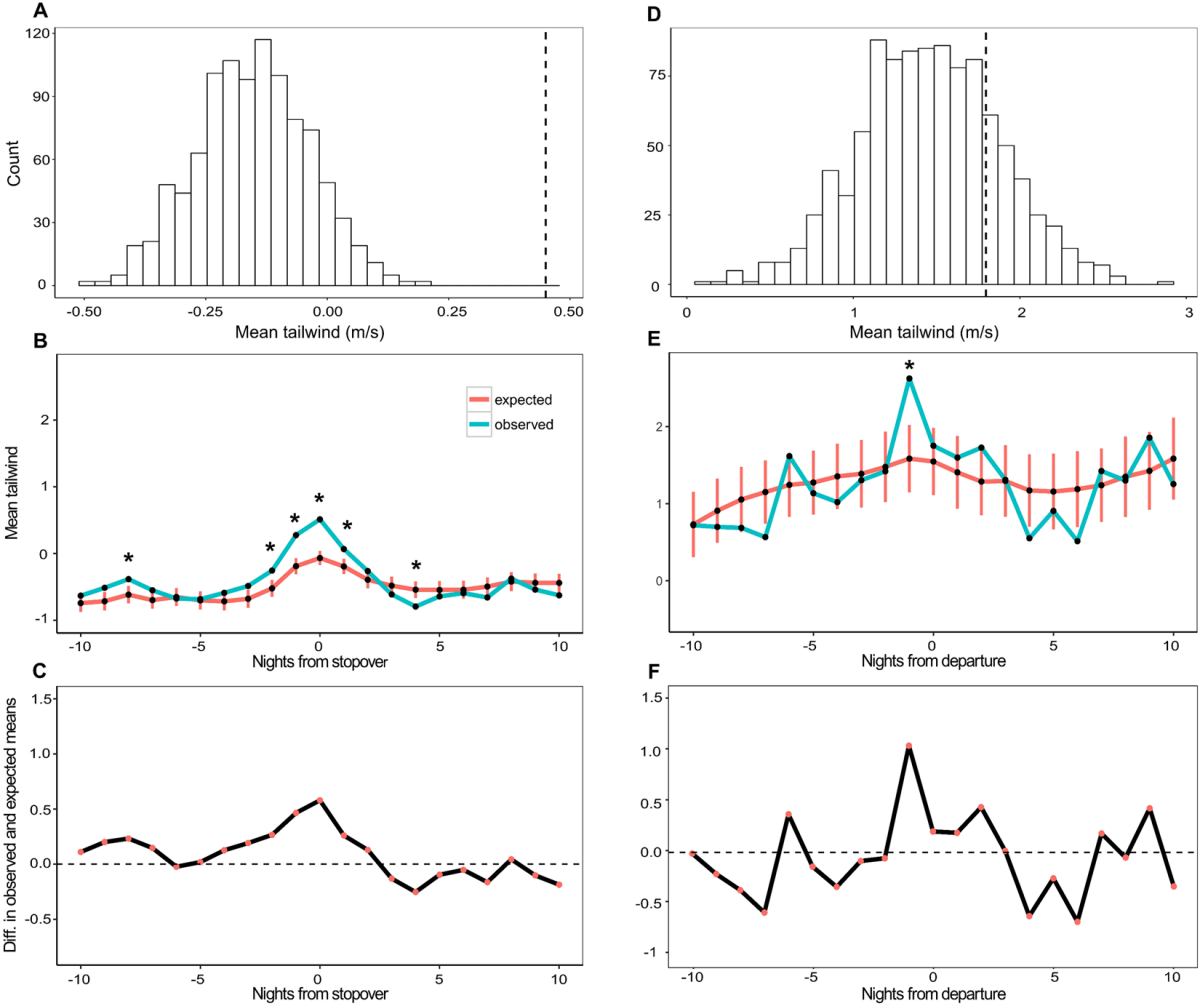
Swainson’s Thrush migrant daily behaviours in a seasonal context. (**A**) The observed mean wind profit on the nights prior to arrival for stopover (dotted line) was significantly better than the expected distribution. (**B**) The two nights immediately before and one night after arrival also had better wind profits than expected. Error bars show standard deviation for expected mean wind profits each night. (**C**) The effect size between observed and expected wind profits was never more than 0.5 m·s^−1^. (**D**) The observed mean wind profit on the nights of departure (dotted line) was no different than the expected distribution. (**E**) The day before departure were significantly different than expected. (**F**) The effect size between observed and expected mean wind profits on the day before departure was 1 m·s^−1^.

Swainson’s Thrushes arrived at Ft. Morgan 497 out of 1074 available nights (46%), and these nights had significantly better wind support than if they had arrived on random nights (mean_expected_ = −0.14 m·s^−1^, n_simulations_ = 1000, p = 0.001; Fig. 4A). Mean tailwinds on arrival nights were better (0.45 ± 3.51 m·s^−1^) compared to nights that they did not arrive (−0.66 ± 3.64 m·s^−1^). The tailwinds were also better than expected on the night before and the night after arrival and worse than expected 4 nights and 10 nights after arrival (Fig. 4B,C).

### Departure decisions

The mean and standard deviation of tailwinds at civil twilight for departure from Ft. Morgan were 0.35 ± 4.56 m·s^−1^. Tailwinds at Ft. Morgan were supportive (i.e., > 0 m/s) for departure 44% of all nights. During the periods experienced by radio-tagged thrushes, Ft. Morgan wind support was 1.46 ± 3.63 m·s^−1^ (n = 147 nights). Probability of departure over the Gulf was not predicted by tailwind, period of the season (“early” or “late”), or their interaction (χ^2^ = 3.70, df = 3, p = 0.30; Table 2); multicollinearity was low (VIF_period_ = 1.27, VIF_tailwind_ = 2.45, VIF_period_ × _tailwind_ = 2.60). None of the predictors were statistically significant.

**Table 2:** Logistic regression covariates of analysis on the probability of departure from Ft. Morgan over the Gulf of Mexico during the period of a radio-tracking study (147 nights), in relation to time of season (period), mean tailwind, and the interaction between period and tailwind.

	Estimate ± S. E	p
Period (Late)	−0.658 ± 0.406	0.105
Mean tailwind	0.023 ± 0.081	0.776
Period × Mean tailwind	0.014 ± 0.105	0.896
Intercept	−0.375 ± 0.317	0.237

**Figure 5:**
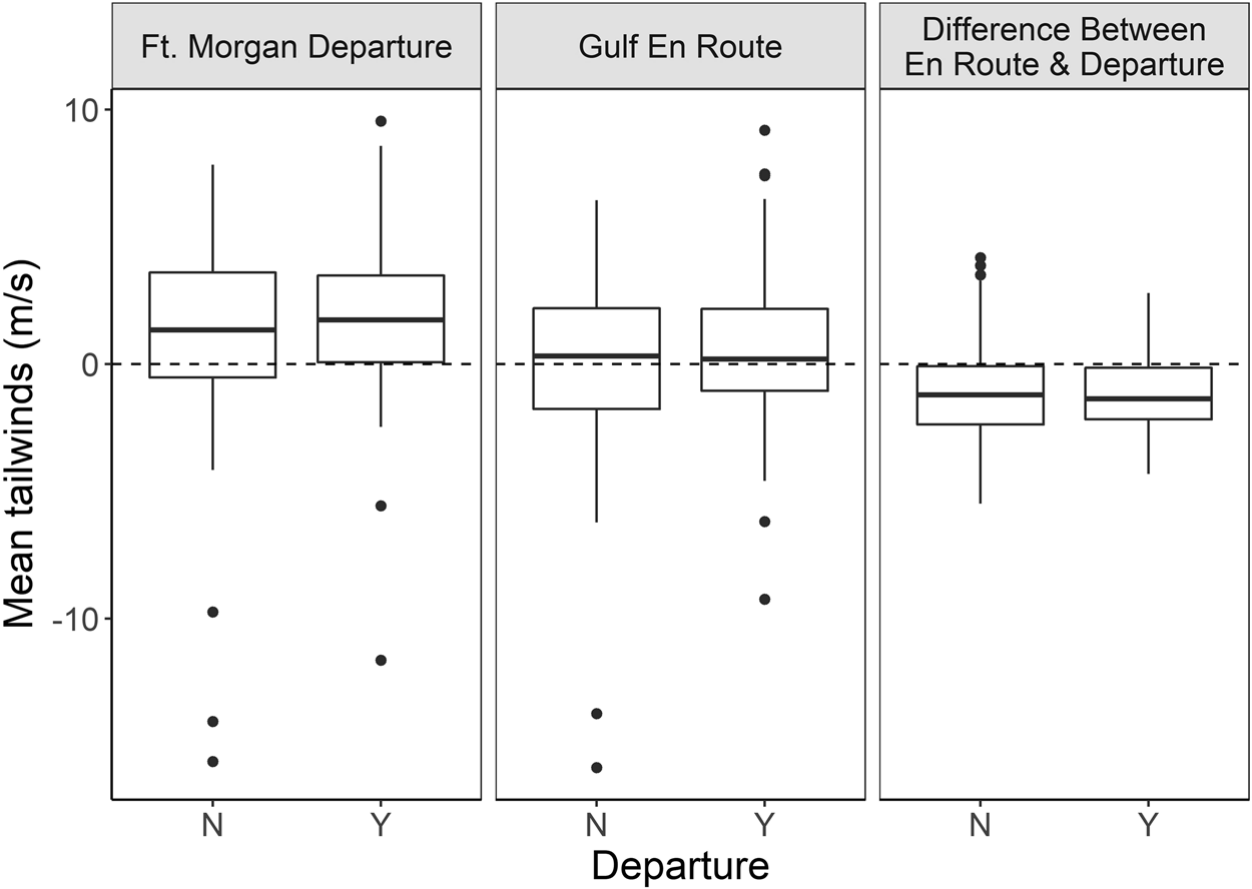
Wind profits on departure and non-departure nights. The greatest effect size was between winds at Ft. Morgan at departure, suggesting the local winds affect departure decisions more than future conditions. Boxplots show actual distributions, with medians and interquartile ranges.

Radio-tagged Swainson’s Thrushes departed over the Gulf 48 out of 147 available nights (33%). The nights that birds chose to depart over the water did not have significantly better wind support for departing from Ft. Morgan (mean_expected_ = 1.46 m·s^−1^, n_simulations_ = 1000, p = 0.22) than randomly chosen nights (Fig. 4D). The nights that birds departed across the Gulf had a mean tailwind of 1.80 ± 3.8 m·s^−1^ at Ft. Morgan upon departure, whereas nights that birds did not depart had winds with a mean tailwind of 1.30 ± 3.4 m·s^−1^ at Ft. Morgan (Fig. 5). Tailwinds were not significantly better or worse for any of the nights before or after departure (Fig. 4E,F).

### Forecasting

**Figure 6:**
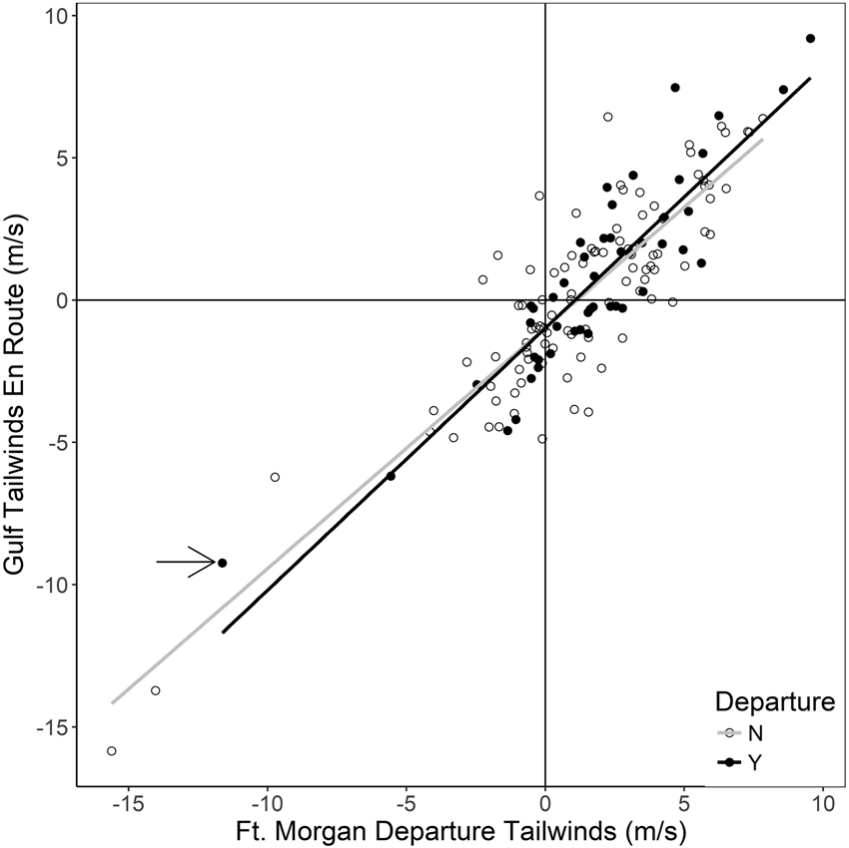
Relationship between nightly winds at Ft. Morgan at departure and mean Gulf-wide wind profits. Winds at Ft. Morgan did predict future winds. The slopes are similar between departure and non-departure nights. Only one bird chose to depart on a night when wind profit was less than −5 m·s^−1^ over the Gulf (arrow), and this bird was not headed toward the longest route across the Gulf, it likely arrived at northern Florida shortly after departure.

Mean tailwinds at Ft. Morgan at departure were worse than those over the Gulf of Mexico; they improved by a mean of 1.15 ± 1.76 m·s^−1^. Ft. Morgan winds were positively correlated with Gulf winds (r = 0.88, df = 145, p < 0.0001; Fig. 6). During the period that thrushes were radio-tagged, nights had consistent wind support (i.e., supportive tailwinds remained supportive and non-supportive headwinds remained non-supportive) 79% of the time (Supplementary Table S1), and 77% of the nights that Swainson’s Thrushes departed had consistent winds. Seventy-four percent of all tracks during the period that thrushes were radio-tagged had consistent winds for the entire crossing, and the mean proportion of each track that had winds consistent with the winds at departure was 0.88 ± 0.24. Of those that changed, winds remained consistent for 13.1 ± 9.9 hours after departure.

Swainson’s Thrushes left on nights that had similar wind support over the Gulf than random available nights (mean_expected_ = 0.31 m·s^−1^, n_simulations_ = 1000, p = 0.213). Gulf tailwinds were 0.13 ± 3.64 m·s^−1^ on departure nights and 0.67 ± 3.46 m·s^−1^ on non-departure nights (Fig. 5). On departure nights, the difference between winds over the Gulf and winds at departure at Ft. Morgan were no better or worse than on random nights (mean expected = −1.15 m·s^−1^, n_simulations_ = 1000, p = 0.45). There was little difference between Gulf and Ft. Morgan tailwinds on departure nights (−1.12 ± 1.55 m·s^−1^) and non-departure nights (−1.17 ± 1.86 m·s^−1^, Fig. 5). There was also no difference in the proportion of the tracks that remained consistent

(i.e., always supportive or always non-supportive) as the winds encountered during departure from Ft. Morgan (mean_expected_ = 0.88, n_simulations_ = 1000, p = 0.44). The proportion of the tracks that remained consistent was 0.88 ± 0.13 on departure nights and 0.88 ± 0.11 on non-departure nights.

### Flight range estimate

According to the Flight models, a medium-sized Swainson’s Thrush^24^ with maximal fuel stores can fly more than 84 hours and 3020 km in still air while maintaining 10 m·s^−1^ airspeed for the entire flight. Of all departure night tracks, the best trans-Gulf tailwind observed was 9.2 m·s^−1^, and the worst was −9.2 m·s^−1^. Were the same medium-sized thrush to fly with a tailwind of 9.2 m·s^−1^ for 84 hours, it could travel approximately 5800 km (ignoring energetic costs of turbulence associated with higher wind speeds), but only 240 km in a headwind of −9.2 m·s^−1^.

Following an optimized airspeed strategy, a thrush could travel approximately 68 hours and 3600 km at 13 m/s. Were the thrush able to fly at this airspeed with a tailwind of 9.2 m·s^−1^ for 68 hours, it would travel 5400 km, but only 930 km in a headwind of −9.2 m·s^−1^.

## Discussion

Migrants on the northern coast of the Gulf of Mexico were equally likely to encounter headwinds than tailwinds during fall migration (consistent with radar observations from 1961–1990^5,25^), but there was huge variation. Given that tailwinds were not uncommon, and headwinds make crossing the Gulf of Mexico more energetically expensive and potentially dangerous, it would be more energetically efficient for birds to stop over when they encounter headwinds and to fly with tailwinds. At the population level and annual scale, the evidence supported the prediction that stopover should occur more often with non-supportive winds. However, at the nightly scale, migrant Swainson’s Thrushes were more likely to arrive for stopover when wind conditions were better than average, although the effect size was very small and there was broad variation in the conditions in which migrants chose to arrive.

The apparent contradiction between annual and nightly time scales is logical when considering movement at a larger spatial scale. Consistent with observed, continent-wide patterns of fall migration in North America^26^, birds are more likely to migrate on nights when synoptic weather systems provide favourable conditions over a large extent, and may not migrate at all when and where conditions at departure are poor. The choice to stop over on the coast is subsequent to the upstream choice to migrate at all on a given night; if birds do not migrate, they cannot arrive for stopover. Therefore, at the nightly scale, wind profits on arrival nights are at least slightly better than the nights when migrants do not move at all. Once aloft, a migrant encountering a coastline may choose to continue over the Gulf without stopping, although these birds would not be captured at a banding station. Thus, if wind support in a given year is better than average, and it takes less energetic stores to successfully cross the Gulf, then a larger proportion of birds can choose to pass over the site and continue across the Gulf. This would result in lower annual capture rates in years with higher mean tailwinds, as observed.

Of the birds that are captured, the decision to stop is likely based on more factors than headwinds alone. One important factor is whether birds are energetically prepared to make a long water crossing^27–29^. A certain proportion of individuals stop on the coast regardless of wind conditions to replenish their energetic stores. In contrast, birds with enough reserves to survive a long flight with headwinds have no reason to stop over: winds are the most relevant to the decision to stop over for birds with moderate energetic reserves^30^. In a given night, the exact amount of energetic reserves needed to cross the Gulf should be related to the magnitude of wind support (i.e., more wind support means that less energetic reserves are needed); therefore, if the distribution of energetic reserves among the population of migrating thrushes is consistent from year to year, fewer thrushes should stopover in years with higher mean wind profits, as the threshold for stopping over due to insufficient energetic stores should be lower. Alternatively, if in a given year migrants have experienced better winds upstream, more individuals might have been able to conserve more energetic reserves prior to encountering the coastline (i.e., the distribution of energetic reserves differs annually), consequently more individuals are able to forgo stopover on the coast.

Variation in the circumstances of arrival for stopover at the coastal site may also account for some variation in selectivity. Whether energetically prepared for crossing or not, migrants may not only arrive and stopover on nights with headwinds, but also arrive on nights with initially supportive winds that deteriorate over the course of the night. In addition, winds the night before and after stopover nights were better than expected, suggesting that good winds are not temporally independent, but occur several nights in a row, as noted in other studies^19^. Thrushes often arrive and stop over with good winds, but the correlation of winds among nights means that a slight delay of a day will not affect the wind support that they will have for crossing. Waiting a day might also be beneficial for migrants that have already made a moderately long flight over land, arriving at Ft. Morgan in the middle of the night (note that daylight itself does not stop these individuals, which take 15–33 hours to cross the Gulf^30^, flying through the night and day). A brief stopover of less than twenty-four hours would allow them to rest and/or forage to refuel prior to departing the next evening without compromising wind support. A brief delay would also allow a migrant to assess local wind conditions, which presumably are more similar to Gulf conditions than those it encountered upstream when it initiated its migratory flight prior to arriving for stopover.

Our prediction that early migrant would be more selective of tailwind than time-constrained migrants traveling in the later part of the season was only supported for birds that were arriving for stopover at Ft. Morgan, although the effect size was modest and there was a lot of variation in winds among arrival nights. Time period was not a significant predictor of the probability of a bird’s departure over the Gulf. Unlike many migrant passerines, Swainson’s Thrushes sometimes hold territories on the wintering grounds^31^. If territorial, individuals benefit from getting to the wintering grounds quicker than competitors, but not so quickly to run out of resources en route. Selection for optimizing this trade-off may result in consistent timing through an endogenous program. Swainson’s Thrushes’ consistent headings and airspeeds^32^ are consistent with an endogenous program, and differs from many other species that adjust both during a flight or at least partially compensate for wind drift^33,34^. If selection for consistent timing is strong, then variation in individuals’ arrival timing may be due primarily to differences in breeding and overwintering locations among individuals^35^.

Overall, the winds that thrushes chose for departure for a Gulf crossing were no more supportive than expected from those available. However, migrant thrushes mostly seemed to avoid the worst conditions. This finding is consistent with the observation that when Swainson’s Thrushes migrate short distances over land they avoid winds greater than 15 m·s^−1^, likely due to the increased energetic demands of flying through higher wind speeds with higher turbulence^36^. Passerines in central Europe were slightly more selective, migrating less frequently when winds exceeded 7 m·s^−1^, but also often accepting moderate headwinds^37^. Furthermore, although migrant thrushes chose the best winds available within a 21-day period to arrive at Ft. Morgan, the best were often weakly supportive or not supportive at all, and migrating Swainson’s Thrushes were often willing to accept weak or moderate headwinds for crossing the Gulf of Mexico, perhaps because the energetic consequences of flying in headwinds were modest enough that they did not affect mortality risk in this species.

Although birds were only weakly selective of winds, they were slightly more selective of winds over land (i.e. arriving for stopover) than over the water (i.e. departure over the Gulf), which is counterintuitive, as water crossings do not have opportunities for landing over relatively long expanses (e.g. 1000–1500 km across the Gulf). Our site at Ft. Morgan is found at 30° N, where the trade wind zone begins. Barring a tropical storm (which are more common in the early part of the season) the trade winds are relatively mild. Despite the inclination to define the Gulf as a barrier to movement, its relatively low winds may mean that it is not a significant barrier for species with strong flight morphologies and individuals of these species with sufficient energetic stores to fuel flight for the distance of the crossing.

One reason that Swainson’s Thrushes can accept headwinds when crossing the Gulf of Mexico is that individuals with sufficient fat stores have the physiological ability to travel over 3000 km in still air: twice as far as the farthest Gulf crossing from Ft. Morgan (1500 km) or equivalent to a direct flight to Columbia across both the Gulf of Mexico and the Caribbean Sea. They could travel even farther with tailwinds. Swainson’s Thrushes would run out of energetic stores if they experienced average headwinds greater than 5 m·s^−1^ while attempting the 1500 km flight to Veracruz with a constant 10 m·s^−1^ airspeed. However, on these nights they could avoid starvation by increasing their airspeed to 15 m·s^−1^ (within the range of observed migratory airspeeds for this species^32^), choosing a shorter trans-Gulf route, or foregoing trans-Gulf migration on any night when headwinds are greater than 5 m·s^−1^. In the radio-tracking period, only 3% of all nights had mean Gulf-wide headwinds greater than 5 m·s^−1^.

To avoid Gulf-wide winds with headwinds greater than 5 m·s^−1^, migrants must be able to forecast them prior to departure. Ft. Morgan winds were mostly predictive of the conditions a migrant would encounter crossing the Gulf. This relationship was strongest at extreme wind speeds, and when wind profits were supportive at departure; winds at lower speeds were less consistent. Despite the relative predictability of winds, there is not much evidence that birds were responding to future wind conditions. The difference between departure and non-departure wind profits at Ft. Morgan at departure was greater than the difference between Gulf-wide winds and between the similarity of Ft. Morgan and Gulf winds, suggesting that current, local winds at Ft. Morgan at departure have the most influence on migrant departure decisions.

Even without relying on precise forecasting, birds could avoid Gulf-wide headwinds greater than 5 m·s^−1^ if they departed only when wind profits at Ft. Morgan were supportive 100% of the time, or 99% of the time if they followed the less conservative rule of avoiding departure when headwinds at departure are greater than 7 m·s^−1^ ^37^. Ninety-eight percent of departing thrushes did avoid these unfavourable nights. The only thrush that chose to depart Ft. Morgan when initial wind conditions were −11.6 m·s^−1^ and mean Gulf-wide wind conditions were −9.2 m·s^−1^ (Fig. 6), left with a departure direction of 130°; leaving at this direction on this night would have resulted in quick landfall in nearby northern Florida.

In summary, migrating Swainson’s Thrushes with sufficient fat stores are well equipped to handle the expanse of the Gulf of Mexico, which has typically moderate wind speeds well within their flight ability. Speculatively, their physiology and wing morphology may have been shaped to withstand Gulf conditions by the pressure to arrive early to secure quality winter territories, as waiting for uncommon supportive winds would not be beneficial. Moreover, the trans-Gulf route has been often discussed as a single stage of migration ending on the southern coastline, but for some South American wintering populations it could be only part of a larger stage across both the Gulf and the Caribbean, and indeed Swainson’s Thrushes often continue migrating past the Yucatan coast^30^, although there is evidence that more arrive in Colombia from Central America than the Caribbean^38^. By following very simple rules of departure, they could ensure their survival at least to the southern Gulf Coast, at which point they could reassess whether to continue or stop over depending on their current condition. In conclusion, the strategies of Swainson’s Thrushes are not necessarily shared by all migrants, or even all passerine migrants. The degree of wind selectivity is likely species specific due to differences in wing morphology, physiology, and behavioural context, as these differences affect the energetic and/or arrival timing consequences of wind selectivity^14,25^.

## Methods

All experimental protocols were approved by the University of Southern Mississippi’s IACUC (Protocol No. 11092210, PHS Animal Welfare Assurance Number A3851-01) and in accordance with the “Guidelines to the Use of Wild Birds in Research” published by the Council of the Ornithological Societies of North America (2010).

### Data

#### Capture data

To assess whether winds affected birds’ decisions to interrupt flight and stop over along the northern coast of the Gulf of Mexico, we used long-term capture data of nocturnally migrating Swainson’s Thrushes at the University of Southern Mississippi’s fall migration bird banding station on the Bon Secour National Wildlife Refuge, Ft. Morgan Peninsula, Alabama, USA (30.2° N, 88.0° W). The banding station was operated daily for approximately four hours starting at sunrise between 1 Sep and 31 October from 1992–2012, except on days when weather was inclement (n = 78 closure days over 21 years; 4.3 ± 3.2 days per year). Thrush capture rates, calculated as the number of thrushes captured per net-hour, provided an index of the number of birds stopping over at the site each night and each fall.

#### Telemetry data

To assess whether winds affected birds’ probability of departure, we used two to three automated radio-telemetry stations (ARTS) to track Swainson’s Thrushes on the peninsula from 2008–2012 to estimate their departure dates, times and directions. Birds were fitted with radio transmitters that had unique frequency signals, and were subsequently tracked at 3–6 minute intervals until the time they departed the Ft. Morgan Peninsula. We calculated the departure date, time, and direction from the last three detections for each bird. When possible triangulation was used to plot birds’ departure tracks and estimate departure directions, otherwise, we estimated birds’ vanishing bearings based on differences in signal strength among the six antennas on the ARTS with the strongest signal (see ref.^30^ for full description of telemetry methods). Individuals departed Ft. Morgan in a variety of directions (α = 133°, µ = 0.136, n = 124), but the analyses in this paper are restricted to individuals that departed over the Gulf of Mexico, in southerly directions, between 105° and 255° (α = 176°, µ = 0.822, n = 62) as these departure directions are consistent with migratory flights across the Gulf. We used telemetry data to assess whether birds demonstrated wind selectively at the time of departure and to evaluate birds’ ability to forecast wind conditions.

#### Wind data

For both the stopover and departure analyses, we used wind data modelled by the National Centers for Environmental Prediction (NCEP), the North American Regional Reanalysis (NARR)^39^, retrieved using Movebank’s Env-DATA service^40,41^. NARR has a temporal precision of 3 hours, a horizontal precision of 32 km, and a vertical precision of 250 m. Specifically, we requested winds data from 1 Sep to 31 Oct, 1990–2012 over the Gulf of Mexico, bounded between 15° to 32° north latitude and 75° to 100° west longitude. Although winds data are available based on pressure level (mb), Env-DATA calculates the N/S (v-wind) and E/W (u-wind) components at heights above the ellipsoid in meters; we requested data at 1 km for both the stopover and departure analyses, an altitude characteristic of typical landbird migration, including over the Gulf^42–44^. Due in part to the vertical precision of this modeled dataset, winds data at different altitudes were correlated across the Gulf of Mexico.

We calculated wind speed and direction by interpolating u-wind and v-wind components using an inverse-weighted distance method, which incorporates data from the nearest four points in the NARR dataset and produces a weighted average to estimate winds at a precise location (e.g., capture/departure location or location along a modelled trajectory). All directions were calculated and described as the direction toward which the wind was going, to make interpretation in relation to bird movement easier; this is in contrast to the convention of meteorologists, who describe wind direction based on the direction from which the wind came.

The focal times for arrival and departure analyses differed. For arrival analyses we estimated the wind speed and direction every three hours (matching the temporal precision of NARR) over the night, specifically at 00:00, 03:00, 06:00, 09:00, and 12:00 UTC. We then calculated the mean and slope across all five time samples to estimate average nightly wind conditions at Ft. Morgan and changes in wind conditions over the course of a night. We calculated a mean value because we do not know the exact time of arrival for individual migrants. For the departure analyses, we used the winds at Ft. Morgan at civil twilight, the time of departure for most nocturnal migrants including Swainson’s Thrushes at this site^29,30^.

For the Gulf forecasting analyses, we developed a forward trajectory model to reflect the dynamic nature of what a small songbird could experience during a trans-Gulf flight using Python 3.3.3. This model simulated typical flight behaviours of small landbird migrants and modelled wind field data to create predicted tracks. The measured variables along these tracks are estimates of the nightly wind conditions experienced by migrants following different trans-Gulf routes, similar to previously published backward trajectory models^10^ Tracks started from Ft. Morgan at civil twilight and moved in one of five potential directions that would move birds toward different endpoints around the Gulf (120°, 150°, 180°, 210°, and 240°; Fig. 1B). For the duration of the simulated flight, modelled birds maintained a constant altitude of 1 km, a constant heading calculated during the first time step, and a constant airspeed of 10 m·s^−1^. These parameters were based on typical altitudes of migrating passerines^42–44^ and the observation that migrating Swainson’s Thrushes maintain their headings and airspeeds (mean observed 10 m·s^−1^) during flight^32^. Although Swainson’s Thrushes are known to make moderate altitude adjustments during flight^45^, these changes in altitude are generally less than the altitudinal precision of NARR (250 m). Heading was calculated as the direction that the axis of a bird’s body needed to be oriented in order to depart in each one of the five directions given wind directions at the time of departure. Airspeed refers to how fast a bird moves in reference to the air, independent of wind support. The behaviours of modelled birds were held constant among tracks, but the predicted routes varied due to the effects of variable winds, which were updated at hourly time-steps. For each time-step, we used inverse weighted interpolation to calculate the estimated wind speed and wind direction at a precise time and location along the track. We then used vector summation of the airspeed/heading vector and wind speed/wind direction vector to determine the groundspeed and track direction, or how fast and in which direction the bird moved relative to the ground. We used the groundspeed to calculate distance travelled over an hour, and used this distance and track direction to determine the next location along the loxodrome, or shortest distance across the Earth’s surface with a constant heading. Simulations continued until the modelled bird reached land or the temporal and spatial extent of the weather data. Temporal extent was defined as a maximum of 45 one-hour time-steps, which was 10 hours greater than the maximal Gulf crossing time observed in Swainson’s Thrushes in this system^30^. We defined the spatial extent of the model using raster data from the land-water mask used by NARR (32 km precision), and we defined “reaching land” as the point on the track when at least one of the closest four surrounding raster points was land.

We used the “tailwind” metric to describe winds each night, at civil twilight and along predicted tracks^46^. Specifically, “tailwind” is the velocity at which the wind is moving the bird in a particular reference direction, where the reference direction usually reflects a bird’s endogenous endpoint or presumed goal; thus, it describes supportive wind direction and speed simultaneously, with larger values indicative of greater wind support and faster movement toward a bird’s endpoint. We defined supportive “tailwinds” as those greater than 0 m/s and unsupportive “headwinds” as those less than or equal to 0 m/s. Tailwind is defined by the following equation:

$${tailwind}=\cos ({\alpha }_{1}-{\alpha }_{2})\,\ast \,{windspeed}$$

where α_1_ equals the direction toward which the bird would continue over the Gulf had it not stopped over (i.e., in this study the five directions encompass a range of potential endpoints around the Gulf as endogenous directions are unknown: 120°, 150°, 180°, 210°, or 240°), and α_2_ equals the wind direction.

For our evaluation of stopover in relation to wind conditions, we calculated average tailwind each night across the five nightly wind values (retrieved at 00:00, 03:00, 06:00, 09:00, and 12:00 UTC) and five directions (120°, 150°, 180°, 210°, or 240°) to account for variability in upstream arrival directions. Parallel movements to the coast are not considered in this study. We excluded nights when the banding station was closed the following day (resulting n = 1076 nights; 1992–2012) to ensure that estimates of wind availability correspond to sampling periods. For the departure analyses, wind profits at Ft. Morgan were calculated as the mean across the five simulated tracks (moving in the five departure directions) for the first time-step; the five simulated tracks always originated at Ft. Morgan at civil twilight. The departure directions of the radio-tracked birds were known but not their actual routes, and a route analysis is beyond the scope of this paper. For the Gulf forecasting analyses, we calculated the mean tailwind of each track by averaging the tailwind for each hourly time step in each simulation and then calculating the mean across tracks, using the same sampling period as for the departure analyses (2008–2012 during the radio-tracking periods).

### Analyses

#### Effect of winds on stopover

To test the effect of winds on the probability of birds stopping over at Ft. Morgan (1990–2012), we correlated annual variation in Swainson’s Thrush capture rates and tailwind. We calculated annual capture rates as the number of captured individuals divided by the total number of net hours for the corresponding year, with one net hour equivalent to one 12 m net open for one hour. Mean annual tailwind was calculated across all nights preceding mornings when nets were opened.

Next we tested whether birds are selective of winds on a given night and whether they are more selective of winds later than earlier in the season. To model the probability of stopover, we used logistic regression (GLM function in the “stats” package of R, using the family = binomial(“logit”) argument) with “stopover night” as the binary response variable, and mean tailwind, “period”, year, and their interactions as the predictors. “Stopover night” (Y = 1, N = 0) was defined by whether or not any Swainson’s Thrushes were captured for the first time the next morning (i.e., to ensure independence, only initial captures of each individual were included in the analysis, not additional recaptures). We considered “stopover night” rather than capture rate as the dependent variable, as the daily capture rate was low and fairly invariable (2.7 ± 3.1 individuals captured/day), and capture rate over the season was non-linear. We transformed Julian date into a binary variable because the relationship between Julian date and capture probability was not linear: “Period” was defined by whether it was “early” (i.e., before the median capture date, dummy coded as “0” by the GLM function) or “late” (i.e., after the median capture date, dummy coded as “1” by the GLM function) in the season. We first ran a full model of all main effects and their interaction. We tested the resultant model’s fit by comparing it to the null model, containing only the intercept as a predictor. Specifically, we computed the chi-square statistic by subtracting the deviance from the null model’s deviance, and computed the p-value from the chi-square distribution and the given degrees of freedom. We calculated the p-value for individual predictors using the z-score of each predictor calculated by dividing the estimate by the standard error.

We used a Monte Carlo (MC) approach to test the prediction that birds stop over following nights with worse than average wind support. We created an expected distribution of mean wind profits by randomly assigning whether a night was an arrival night from the full distribution of nights equal to the observed number of nights that birds stopped at the site 1000 times (using the sample function in R). For each randomly selected sample, we calculated the mean tailwinds of “stopover” nights, resulting in the expected distribution of these 1000 means. The mean of each sample was used to create an expected wind profit distribution, which was compared to the observed mean tailwind across years. The expected tailwind distribution represented average wind support on available stopover nights, or wind support that birds would encounter if they stopped over on a random night. By comparing tailwind on nights when thrushes were observed stopping to tailwind on available nights we were able to assess whether birds chose to stop on nights with worse, or better, wind support than average. We calculated the p-value as (r + 1)/(n + 1), when r was the number of times the expected mean was greater than the observed mean (or less than the observed mean when the observed mean was less than the mean of expected means), and n was equal to the number of simulations^47^.

We also used a Monte Carlo approach to evaluate seasonal changes in supportive wind availability and selectivity. Specifically, we examined the temporal context of stopover choices at both the daily scale and within the context of the 21 days surrounding arrival (±10 days from arrival for stopover). We calculated observed and constructed expected means of tailwind for arrival nights and for up to ten nights preceding and following the focal night. Our sampling period was from 1 September to 31 October; we only sampled arrival nights between 10 September and 21 October, so that every night would be equally represented across the analysis; therefore, the expected and observed means on the night of stopover are slightly different than the full sample MC. Reducing the temporal sampling period by 20 days still included 94% of all captures and 90% of all stopover nights. For the observed curve, stopover nights were defined as those where at least one Swainson’s Thrush was captured the following morning. The tailwinds for ± x nights before and after the observed stopover were also calculated and reported, with x ranging from one to ten nights. For the expected curve, we randomly picked which nights were stopover nights (equal to the number of observed stopover nights) and calculated the mean wind conditions for the randomly chosen “stopover night” and the nights surrounding it, the same way that we calculated the observed values. We drew a random sample of stopover nights 1000 times to create the expected distribution for each of the 21 observed/expected contrasts. Because the measurement window widened and shifted for each night before/after stopover (Supplementary Fig. S2), the curve of expected means reflects seasonal changes in supportive wind availability. We calculated a p-value for each of the 21 observed/expected contrasts using the same MC method previously described.

#### Effects on wind on probability of departure of radio-tagged birds

We used the same methods as for the arrival analysis (logistic regression and Monte Carlo analysis), except we used departure tailwinds (i.e., those at Ft. Morgan at civil twilight) as the predictor variable rather than tailwinds the night preceding capture. Only nights between first capture and last departure of southbound (105°–255°) radio-tagged birds were included (n = 147 nights; 2008–2012), to account for any tagging bias. In the latter MC analysis, reducing the sampling period by 20 days still included 94% of departing birds and 92% of the departure nights.

#### Ability to forecast wind over Gulf based on departure conditions

For the Gulf forecasting analyses, we calculated several metrics to quantify the predictability of winds encountered when crossing the Gulf based on local winds experienced during departure from Ft. Morgan. First, to compare departure conditions to the trans-Gulf conditions, we correlated Ft. Morgan and Gulf tailwinds and then calculated the mean difference between them. Second, we tabulated how often initial supportiveness at departure predicted Gulf-wide supportiveness en route across all nights, departure nights, and non-departure nights. Third, we calculated how long in hourly time-steps it took for each track to change sign (e.g., from supportive tailwinds to unsupportive headwinds), if at all. And fourth, we calculated the proportion of each track that had the same supportiveness as the initial tailwind metric at Ft. Morgan at departure.

We used Monte Carlo tests to test three predictions about Swainson’s Thrushes’ use of future wind conditions to make departure decisions. Specifically, we tested whether migrants choose nights to depart when any of the following were better than expected given available winds: 1) Gulf tailwinds, 2) the difference between Ft. Morgan and Gulf tailwinds and 3) the proportion of tracks that are consistent with the supportiveness (i.e., supportive or not) of winds at departure. All statistical tests were performed in R 3.0.2. Each test was one-tailed, and had an α level of 0.05.

### Estimating flight range

To estimate the flight range of a typical Swainson’s Thrush to evaluate the need for wind selectivity in this system, we used the Migrate module in the program Flight 1.24^24^. This module uses biomechanical and physiological models to predict how long a bird can fly before depleting its energetic reserves. We set most of the parameters using the pre-sets for continuously flapping passerines. We did customize the wing morphology parameters using measurements made on standardized photographs of the right wings of 27 of the Swainson’s Thrushes radio-tagged between 2009–2012. We took 2–3 pictures of each thrush’s wing and back, outstretched according to Flight instructions^24^. We then had three observers independently assess which was the “best” picture according to the Pennycuick criteria and picture quality. Pictures with at least two observers’ agreement were chosen for measurement. We next used ImageJ to calibrate the photo using a ruler in the picture, and then to measure the semi-span and wing area including the root box^24^. We used the semi-span to calculate the wingspan. Each measurement was taken 3 times and the mean of these measurements used to calculate the final mean for Swainson’s Thrushes. This mean wingspan was (0.284 ± 0.016 m) and the mean wing area was (0.015 ± 0.016 m^2^), similar to the wing measurements reported for migrating Swainson’s Thrushes in Illinois^48^. The fat fraction was set to the maximal fat loads of dissected Swainson’s thrushes collected en route from tower kills on the northern Florida Gulf Coast^49^, which is equal to a 0.33 fat fraction of an average-sized thrush (12.3 g fat per 37.3 g total body mass). Lastly, we set the flight altitude to 1000 m ASL and used a constant airspeed of 10 m·s^−1^ to match our model, which is not an optimal strategy. Optimally, individuals would adjust their airspeeds to minimize work depending on mass, which gradually changes as energetic stores are depleted over the duration of the flight. We ran the module again with optimal, dynamic airspeed pre-sets to determine airspeed and flight ranges for comparison, which ranged from 11.2–15 m/s during the simulation.

### Data Availability

All data and associated scripts are available upon request.

## Electronic supplementary material

Supplementary Information
