# Supplementary material for: Swainson’s Thrushes do not show strong wind selectivity prior to crossing the Gulf of Mexico
Source: Sci Rep. 2017 Oct 27;7:14280. doi: 10.1038/s41598-017-14668-3 (PMC5660249; doi:10.1038/s41598-017-14668-3)
Supplement: Supplementary file 1 — Supplementary Information [file 41598_2017_14668_MOESM1_ESM.pdf]

# Swainson's Thrushes do not show strong wind selectivity prior to crossing the Gulf of Mexico

**Rachel T. Bolus[1,2,3,*], Robert H. Diehl[1], Frank R. Moore[4], Jill L. Deppe[5], Michael P. Ward[2], Jaclyn Smolinsky[4,6], and Theodore J. Zenzal, Jr.[4]**

[1]U. S. Geological Survey, Northern Rocky Mountain Science Center, Bozeman, MT 59715, USA
[2]University of Illinois at Urbana-Champaign, Department of Natural Resources and Environmental Sciences, Urbana, IL 61801, USA
[3]Southern Utah University, Department of Biology, Cedar City, UT 84720, USA
[4]The University of Southern Mississippi, Department of Biological Sciences, Hattiesburg, MS 39406, USA [5]Eastern Illinois University, Department of Biology, Charleston, IL 61920, USA
[6]University of Delaware, Department of Entomology and Wildlife Ecology, Newark, DE 19716, USA
[*]rachelbolus@suu.edu

## Supplementary Information

**Figure S1. Migrating Swainson's Thrushes capture phenology.** The median date of capture was 3 October. 50% of all birds were captured within a 12-day window around the median.

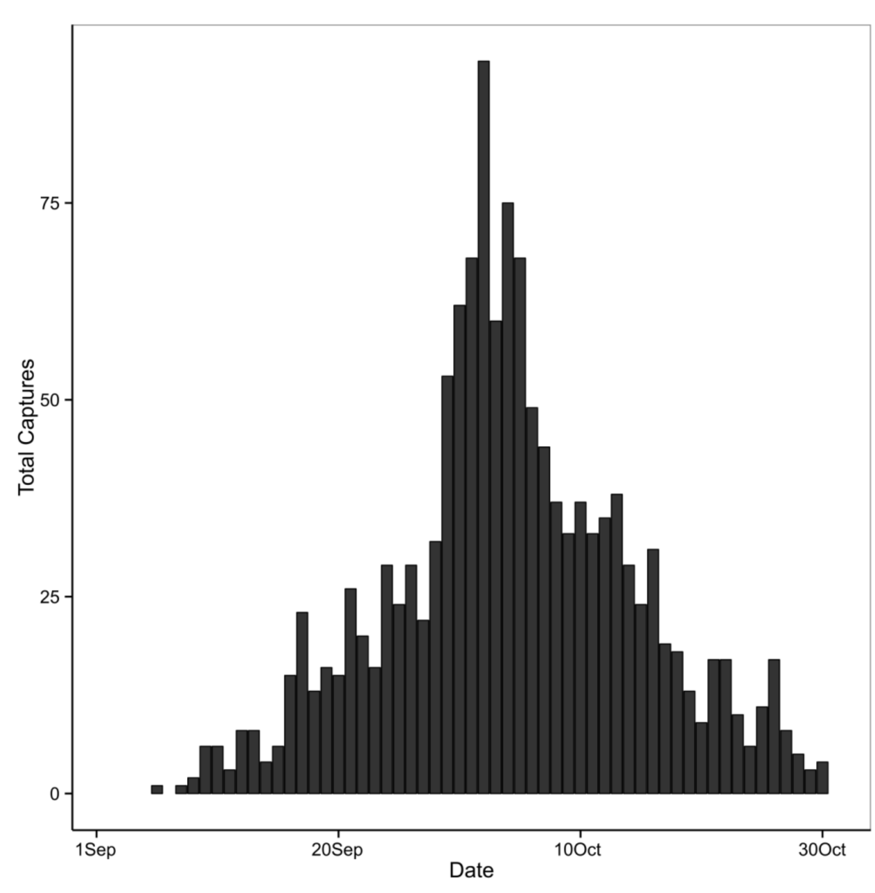

**Figure S2. Sampling scheme for Monte Carlo Analysis.** For the analyses where the arrival for stopover and departure decisions were examined in the context of +/- 10 nights, we sampled only birds between 10 Sep and 21 Oct (represented by the white boxes at 0 nights from arrival for stopover/departure). In the mean wind profit estimates for the nights previous to departure, the measurement window shifted to x nights earlier. Similarly, in the mean wind profit estimated for the nights after departure, the measurement window shifted to x nights later. Therefore, the expected curve in this analysis reflects seasonal changes in availability.

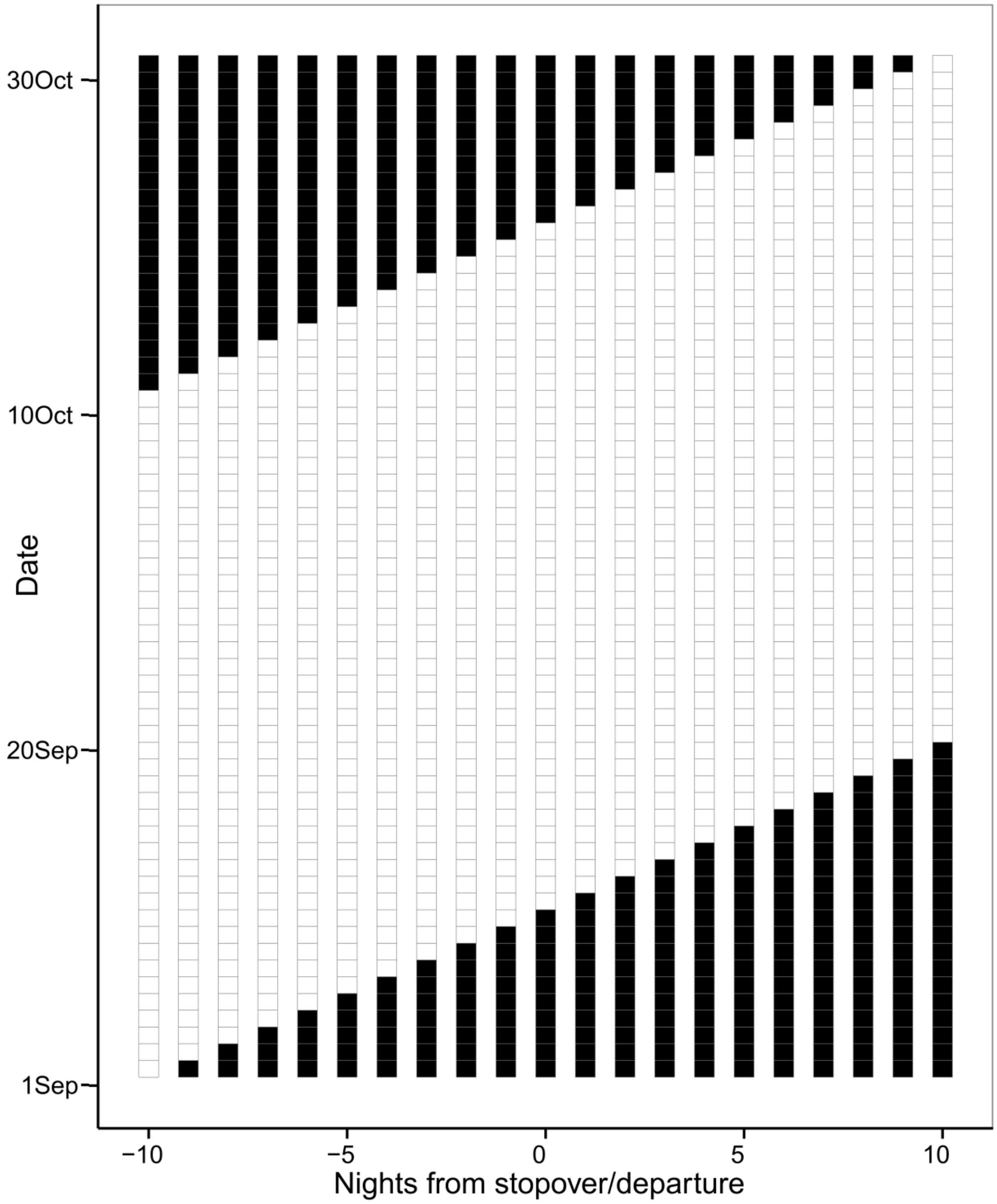

*Table S1: Proportion of nights with consistent and variable winds by initial wind supportiveness*

| **Consistency** | **Ft. Morgan Departure** | **Gulf En Route** | **All Nights** | **Departure Nights** | **Non-Departure Nights** |
|---|---|---|---|---|---|
| Consistent | Supportive | Supportive | 0.503 | 0.521 | 0.495 |
| Consistent | Non-Supportive | Non-Supportive | 0.286 | 0.250 | 0.303 |
| Variable | Supportive | Non-Supportive | 0.177 | 0.000 | 0.051 |
| Variable | Non-Supportive | Supportive | 0.034 | 0.229 | 0.152 |
